# Observation of Distinct Two-Photon Transition Channels in CdTe Quantum Dots in a Regime of Very Strong Confinement

**DOI:** 10.3390/ma10040363

**Published:** 2017-03-30

**Authors:** Marcelo Gonçalves Vivas, José Carlos Leandro De Sousa, Leonardo De Boni, Marco Antônio Schiavon, Cleber Renato Mendonca

**Affiliations:** 1 Instituto de Ciência e Tecnologia, Universidade Federal de Alfenas, Poços de Caldas, Minas Gerais 37715-400, Brazil; 2 Instituto de Física de São Carlos, Universidade de São Paulo, São Carlos, São Paulo 13560-970, Brazil; deboni@ifsc.usp.br; 3 Departamento de Ciências Naturais, Universidade Federal de São João del-Rei, São João del-Rei, Minas Gerais 36301-160, Brazil; jclsousa27@gmail.com (J.C.L.D.S.); schiavon@ufsj.edu.br (M.A.S.)

**Keywords:** CdTe quantum dots, 2PA transition channels, femtosecond two-photon spectroscopy

## Abstract

We report here on the direct observation of distinct two-photon transition channels in glutathione-capped (GSH) CdTe quantum dots (QDs) in a very strong confinement regime. CdTe-GSH QDs with different average diameters (2.5, 3.0, and 3.3 nm) were synthesized through the one-pot method and their two-photon absorption (2PA) spectrum determined by a femtosecond wavelength-tunable Z-scan. Our results show that the two lower-energy one-photon-allowed excitonic transitions, 1S_3/2_(h) → 1S(e) and 2S_3/2_(h) → 1S(e), are also accessed via 2PA. These results were ascribed to the relaxation of the parity selection rules due to the noncentrosymmetric structure of the CdTe QDs (zinc-blended structure), whose magnitude are determined by surface defects and structural irregularities present in CdTe-GSH QDs, in the strong confinement regime.

## 1. Introduction

Semiconductor quantum dots (QDs) are nanomaterials represented by a three-dimensionally confined electron-hole system. Such tight spatial confinement provides interesting optical features, such as size-tunable absorption and emission, which are closely associated with new technologies spanning from biology to physics [1,2,3,4,5,6,7,8,9,10,11,12,13,14,15].

The optical properties of QDs are described by quantum mechanics, being hence subjected to its selection rules [16,17]. Therefore, to describe the selection rules and to unveil the electronic structure of these materials, it is necessary to know the wave-functions symmetry, which defines the parity of the electronic states. The symmetry of each electronic state is expressed by their quantum numbers (principal (*n*), azimuthal (*l*), and magnetic (*m_l_*)), which dictates the electron-hole recombination induced by single or multi-photons absorption. Materials exhibiting inversion symmetry, such as PbS and PbSe QDs, present antagonistic dipole electric allowed transitions induced by one-photon absorption (1PA) and two-photon absorption (2PA), i.e., one-photon-allowed states are forbidden by 2PA and vice versa. Therefore, while the electron-hole recombination excited by 2PA occurs between states satisfying $\Delta l=l_{e}-l_{h}=\pm 1$ (subscript *e* and *h* corresponds to electron and hole, respectively), in one-photon-induced transitions, it occurs only if $\Delta l=l_{e}-l_{h}=0$ [16,17]. Some mechanisms, however, can break the inversion symmetry of the wave functions that describe the electronic states. For example, interactions with a solvent, the Stark transient effect, a magnetic field, surface defects, and structural irregularities, among others, may prevent the wave function from presenting a well-defined parity [17,18,19,20,21]. In this context, one-photon-allowed states may also be accessed via 2PA. However, it is worth mentioning that 2PA-allowed transition probability is strongly dependent on the state symmetry degree. Therefore, broadband analysis of the 2PA spectrum in QDs can provide important information about the electronic and structural features of such nanomaterials.

II–VI semiconductor quantum dots have been shown to present a high 2PA cross section (expressed in GM = 10^−50^ cm^4^ s^−1^ photon^−1^) along the visible and near-infrared regions. Among them, it is worth highlighting ZnS (~200 GM at 600 nm, diameter = 3.8 nm [22]), CdS (4.4 × 10^3^ GM at 800 nm, diameter = 4.45 nm [23]), CdSe (2 × 10^4^ GM at 950 nm; diameter = 3.7 nm [24]), CdTe (~3.0 × 10^3^ GM at 900 nm, diameter = 4 nm [25]), and PbS (6 × 10^4^ GM at 1460 nm, diameter = 3.7 nm [26]) QDs.

**Figure 1:**
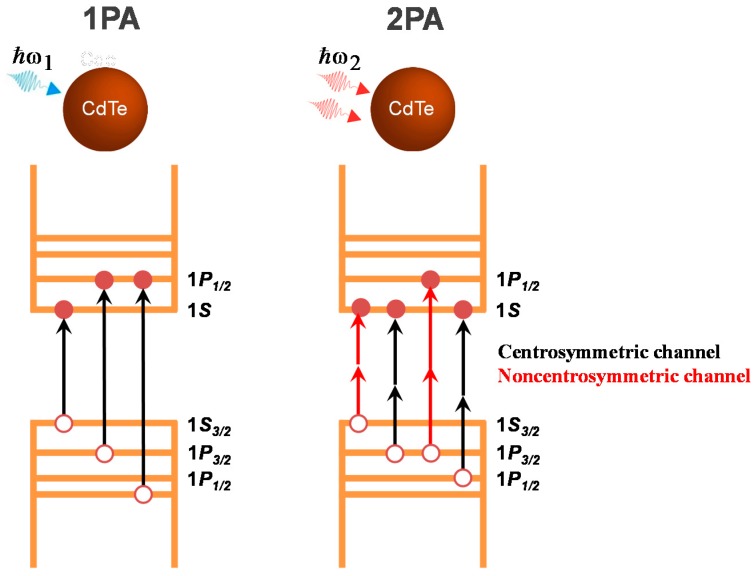
Energy diagram for the lowest energy 1PA- and 2PA-allowed transitions for quantum dots (QDs).

CdTe QDs with a zinc-blended structure (cubic T_d_ structure), as the ones studied here, are noncentrosymmetric semiconductors [27]; therefore, to model the entire 2PA spectra, an additional transition channel should be taken into account, in which the quantum numbers are conserved analogously to the one-photon-allowed transitions, i.e., Δ*l* = 0, the so-called noncentrosymmetric 2PA transition channel. Figure 1 illustrates the energy diagram for the lower-energy 1PA- and 2PA-allowed transitions for CdTe QDs. 

Fedorov et al. [16] deduced an analytical expression for the 2PA cross section containing centrosymmetric and noncentrosymmetric channels, by using the parabolic effective mass approximation. Although this model describes reasonably well the 2PA spectrum for CdS and CdSe QDs in the regime of intermediate confinement, as reported in Refs. [16,22,28], it fails to describe the 2PA-allowed optical transition when CdTe QDs are in a very strong confinement regime, as shown in Ref. [25]. The discrepancy between the experimental and theoretical (Fedorov’s model) data is even higher for CdTe QDs due to its effective mass values for electron and holes (light, heavy, and split-off). Furthermore, the parabolic effective mass approximation presents some limitations: (i) the zero-order approximation of the Hamiltonian for regions where $\vec{k}$ ~ 0; (ii) the fact that the model does not consider mixing among the heavy- and the light-hole bands; (iii) the fact that the effective masses are considered constants (parabolic bands) [29].

Although some studies reported 2PA properties of CdTe QDs [25,29,30,31,32], few present nonlinear spectra in a broad spectral region using femtosecond laser pulses. Furthermore, works on the 2PA in CdTe QDs do not mention the possibility of a contribution from the noncentrosymmetric channel to the 2PA cross section in the very strong confinement regime. For example, Padilha et al. [32] reports on the noncentrosymmetric channel for the 2PA cross section of CdTe QDs in glass matrices in the intermediate confinement regime (diameter higher than 6 nm, Bohr radius for CdTe is 7.5 nm [29]). Even so, the reported degenerate 2PA spectrum, for CdTe QDs does not include the region for the lowest energy transition (1S_3/2_(h) → 1S(e)), and the available data are limited to specific wavelengths. In the same way, Qu et al. [25] reported a spherical eight-band Pidgen and Brown model that considers the mixing between the conduction and the valence bands, as well as the complex structure of the valence bands. However, this model exhibits only 2PA transitions that are governed by the electric-dipole selection rules; therefore, it does not consider the “forbidden” 2PA transition, such as the 1S_3/2_(h) → 1S(e) lowest-energy excitonic transition. In this context, this work reports on the femtosecond 2PA cross-section spectra of water soluble colloidal CdTe-GSH QDs in the very strong confinement regime, emphasizing the influence of the noncentrosymmetric 2PA transition channel. For that, we reported the broadband 2PA cross-section spectra from 600 nm to 1220 nm, in 10 nm intervals, providing high spectral resolution to the nonlinear measurements.

## 2. Results and Discussion

**Figure 2:**
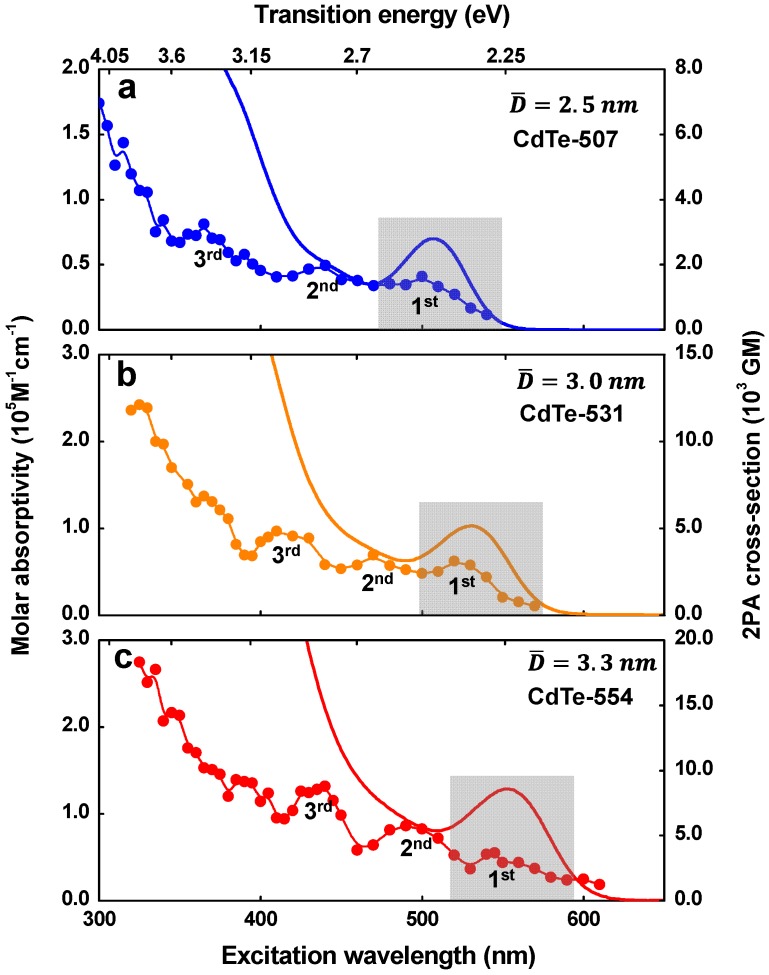
One-photon absorption (1PA) (solid lines, left axis) and two-photon absorption (2PA) (symbols, right axis) spectra for CdTe-GSH QDs (**a**) CdTe-507; (**b**) CdTe-531 and (**c**) CdTe-554. The standard deviation for the 2PA cross section is about 10%.

Figure 2 depicts the one- and two-photon absorption spectra for three CdTe-GSH samples with different average diameters ($\bar{D}$), within the regime of strong quantum confinement, namely CdTe-507 ($\bar{D}=2.5\;nm$), CdTe-531 ($\bar{D}=3.0\;nm$), and CdTe-554 ($\bar{D}=3.3\;nm$). The 1PA spectra (solid lines, left axis) exhibit the well-defined first 1S_3/2_(h) → 1S(e) excitonic transition (lowest energetic band). This transition undergoes a red-shift of approximately 200 meV when CdTe-507 is compared to CdTe-554, indicating an increase in QD size [33]. By using the Yu’s formula for CdTe QDs [34], the average diameter for each CdTe-GSH QDs is estimated to be 2.5 nm, 3.0 nm, and 3.3 nm. CdTe-GSH QDs optical properties, such as molar absorptivity (ε_1PA_), fluorescence maximum position (FMP), fluorescence lifetime (τ_f_), relative fluorescence quantum yield (φ_f_), as well as parameter obtained from the 2PA spectra are shown in Table 1. It is important to mention that the QDs samples exhibit spherical shapes and size dispersion of approximately 25 %, as determined by Transmission Electron Microscopy analysis.

In Figure 2, the symbols along the line (right axis) display the 2PA cross section for CdTe-GSH QDs as a function of half of the excitation wavelength, to provide better comparison with the 1PA. As it can be seen, the 2PA spectra present three defined peaks as labeled on the figure, which are superimposed by the intermediate state resonance enhancement effect (ISRE), described by the monotonic decrease on the 2PA cross section from the UV to the red region. In the ISRE UV region, the 2PA cross section reaches extremely high values, from 6.0 × 10^3^ GM (CdTe-507) up to ~1.8 × 10^4^ GM (CdTe-554). It occurs because the excitation photon energy approaches the first 1PA-allowed transition, increasing the 2PA cross section. [26] Moreover, the higher number of excited states contributing to the 2PA process also increases the 2PA cross section as the excitation energy increases [16]. One can observe that, in the lowest energy 2PA peaks, labeled as 1st in Figure 2, 2PA cross-section values range from 1.6 × 10^3^ GM (CdTe-507—Figure 2a) to 2.9 × 10^3^ GM (CdTe-554—Figure 2c). Moreover, an important feature of this state, observed in this work, is that it is allowed by one- and two-photon absorption, indicating that the 1S_3/2_(h) → 1S(e) transition has its electric-dipole selection rules relaxed.

**Figure 3:**
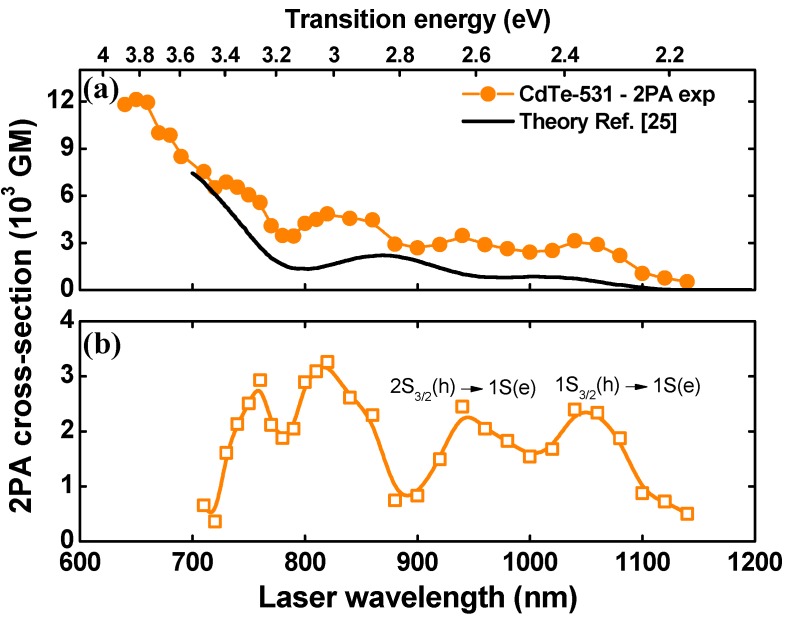
(**a**) Experimental (dots) and theoretical (solid line, data from the Ref. [25]) 2PA spectrum for CdTe-GSH QDs (CdTe-531); (**b**) The difference between the experimental and theoretical 2PA spectrum reported in part (**a**).

A few years ago, the 2PA cross-section spectra for CdTe QDs were investigated in Refs. [25,29] using, respectively, the $\vec{k}\cdot \vec{p}$ model, including the mixing among the heavy- and light-hole bands, and the spherical eight-band Pidgen and Brown model [27], which considers the mixing between the conduction and the valence bands as well as the complex structure of the valence bands. However, in both studies, the correspondence between 1PA and 2PA transitions was not observed, indicating a discrepancy with our experimental results. In order to visualize it, in Figure 3a, we compare the experimental 2PA spectrum we obtained (dots) for the CdTe-531 (*D* = 3.0 nm) with theoretical data obtained from Ref. [25] (solid line).

Based on Figure 3a, two important differences between the experimental and theoretical spectrum can be highlighted. The first one is that the 2PA cross sections for the experimental data are slightly higher than theoretical ones. The second one is that the experimental 2PA spectrum presents more 2PA transitions as compared to the theoretical data. Moreover, such two aspects are closely related because the increase in the number of transitions tends to enhance the 2PA cross section. At the same time, both differences should be associated with the noncentrosymmetric 2PA transition channel due to cubic zinc-blended structure with T_d_ symmetry of the CdTe QDs. To aid in the understanding of these important outcomes, in Figure 3b, the difference between the experimental and theoretical 2PA spectrum were plotted, which allows for the obtainment of information about the noncentrosymmetric 2PA transitions. Proceeding in this way, one can note that the two lower-energy 1PA-allowed transitions, i.e., the 1S_3/2_(h) → 1S(e) (peak at 2.32 eV) and 2S_3/2_(h) → 1S(e) (peak at 2.62 eV), are observed along to the 2PA spectrum, corroborating our previous analysis. Higher energy transitions are also observed in Figure 3b, but they cannot be separately identified because of the large number of transitions in this spectral region.

Therefore, these results confirm that parity selection rules were relaxed for the CdTe-GSH QDs samples, presented here. Another interesting feature that can be observed in Figure 2 is that the 2PA strength for the 1S_3/2_(h) → 1S(e) transition normalized by the QD volume decreases with larger QDs. More specifically, the 2PA figure of merit (FOM), defined as the 2PA cross sections divided by the QD volume, decrease from 196 GM/nm^3^ ($\bar{D}=2.5\;nm$) to 154 GM/nm^3^ ($\bar{D}=3.3\;nm$).

This result is attributed to a decrease in surface defects and improvement of the structural regularity for larger CdTe-GSH QDs. In fact, the fluorescence quantum yield for these samples rise as a function of QD size (see Table 1) due to a better QDs surface quality ascribed to the Ostwald ripening mechanism [35]. In this process, an increase in the synthesis time causes the dissolution of smaller QDs, which precipitate onto the surface of larger QDs. As a consequence, the average QD size increases causing an increase in the surface quality and structural regularity of nanocrystals.

In Figure 2, the 2PA spectra exhibits two higher-energy peaks, labeled as 2nd and 3rd, with a 2PA cross section ranging from 1.97 × 10^3^ to 5.65 × 10^3^ GM. The magnitude of these 2PA peaks are in the same order of those published in Ref. [25,29,32], indicating that the 2PA cross section in these regions are predominantly related to higher energy 2PA-allowed excitonic transitions. However, as shown in Figure 3b, a considerable contribution of the noncentrosymmetric 2PA transition channel was observed for the higher energy region of the 2PA spectrum. It is interesting to note that the FOM for the 2nd and 3rd maxima increase with larger QDs, i.e., FOM^2nd^ = 245 GM/nm^3^ and FOM^3rd^ = 322 GM/nm^3^ for CdTe-507 and FOM^2nd^ = 300 GM/nm^3^ and FOM^3rd^ = 454 GM/nm^3^ for CdTe-554. This behavior corroborates our previous results because a reduction of surface defects leads to a decrease in the 2PA forbidden transition strength (as pointed by the FOM for the 1S_3/2_(h) → 1S(e)), while it increases for 2PA-allowed transition, analogous to what occurs in organic chromophores [20,36].

## 3. Materials and Methods

We used the one-pot method, whose details can be found in Refs. [37,38], to synthesize glutathione-capped CdTe QDs. The linear and nonlinear optical measurements were performed in aqueous solutions of GSH-capped CdTe QDs, with concentrations on the order of 10^16^ QDs/cm^3^ and 10^17^ QDs/cm^3^, respectively. The steady-state absorption and fluorescence spectra were recorded using a Shimadzu UV-1800 spectrophotometer (Shimadzu, Kyoto, Japan) and a Perkin Elmer LS55 fluorimeter (Waltham, MA, USA), respectively.

The fluorescence quantum yields (φ_f_) of the nanocrystals were determined using the fluorescence spectrum of the samples, in a comparative method that uses the fluorescence spectrum and quantum yield of a reference sample [39]. Here, we used Rhodamine 6G dissolved in water as the standard fluorescent dye (φ_f_ = 92%).

Fluorescence lifetime was measured by exciting the CdTe QDs at 532 nm (frequency double of a Q-switched and mode-locked Nd:YAG—70 ps). The 532 nm beam was focused into the sample, placed in a 2-mm-thick fused silica cuvette, with a lens with a focal length of 12 cm. The fluorescence signal was collected perpendicularly to the excitation beam by an optical fiber positioned close to the fluorescent spot. The signal was acquired by a silicon photodetector with a rise time of approximately 0.5 ns and subsequently averaged and recorded with a digital oscilloscope (5 GS/s).

## 4. Final Remarks

The relaxation of the parity selection rules in noncentrosymmetric CdTe-GSH QDs at the very strong confinement regime was observed. Our results show a coincidence between 1PA and 2PA peaks, indicating that a one-photon transition is also allowed by two-photon excitation. To explain this, we considered that, due to the zinc-blended structure with T_d_ symmetry, CdTe QDs do not present inversion symmetry; therefore, the parity of the electronic states involved in optical transitions are not precisely defined. Thus, the coincidence between the lowest energy 1PA and 2PA peaks can be explained through the relaxation of the parity selection rules. In addition, we show that the reduction in surface defects and structural irregularities with the increase in QD size, very common in colloidal QD synthesis in the strong confinement regime, increases the centrosymmetric and decreases the noncentrosymmetric 2PA channel strength.

## Figures and Tables

**Table 1:** Optical properties of CdTe-GSH QDs.

$\bar{D}\;\left(nm\right)$	1PA Peak (eV)	ε (10^5^ M^−1^cm^−1^)	φ_f_	FMP (eV)	τ_f_ (ns)	2PA Cross-Section (10^3^ GM)	FOM (GM/nm^3^)	2PA Transition (eV)
2.5	2.45 (507 nm)	0.70	0.13	2.27 (547 nm)	35	1^st^ → 1.60 2^nd^ → 1.97 3^rd^ → 3.24	196 240 396	2.45 2.82 3.40
3.0	2.34 (531 nm)	1.01	0.16	2.15 (576 nm)	43	1^st^ → 3.12 2^nd^ → 3.46 3^rd^ → 4.55	220 245 322	2.34 2.64 3.00
3.3	2.24 (554 nm)	1.27	0.29	2.07 (599 nm)	50	1^st^ → 2.92 2^nd^ → 5.65 3^rd^ → 8.54	155 300 454	2.24 2.50 2.85
